# Large area flexible pressure/strain sensors and arrays using nanomaterials and printing techniques

**DOI:** 10.1186/s40580-019-0198-x

**Published:** 2019-09-09

**Authors:** Chithra Parameswaran, Dipti Gupta

**Affiliations:** Plastic Electronics and Energy Laboratory, Department of Metallurgical Engineering and Materials Science, Indian Institute of Technology Bombay, Mumbai, 400076 India

**Keywords:** Elastomer, Sensor, Piezocapacitance, Piezoresistance, Patterning, Printing, Embedded printing, Textile sensors, Nanomaterials, Wireless sensing

## Abstract

Sensors are becoming more demanding in all spheres of human activities for their advancement in terms of fabrication and cost. Several methods of fabrication and configurations exist which provide them myriad of applications. However, the advantage of fabrication for sensors lies with bulk fabrication and processing techniques. Exhaustive study for process advancement towards miniaturization from the advent of MEMS technology has been going on and progressing at high pace and has reached a highly advanced level wherein batch production and low cost alternatives provide a competitive performance. A look back to this advancement and thus understanding the route further is essential which is the core of this review in light of nanomaterials and printed technology based sensors. A subjective appraisal of these developments in sensor architecture from the advent of MEMS technology converging present date novel materials and process technologies through this article help us understand the path further.
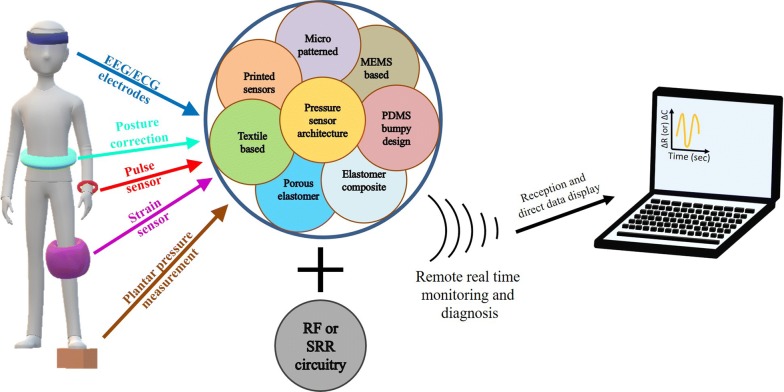

## Introduction

Sensors have become an inevitable part of human needs for their immense participation in all spheres of activities from daily lives to all job sectors. These appear in one or other forms, directly or indirectly guiding us in time. Looking into their development from inception at this point is necessary to understand the manifold evolution in its sensing mechanism and materials involved. This review is intended to take us through the milestones that have rendered sensors their indispensable role in this living platform. We provide the basic understanding of the sensor and proceed towards the highly mature sensor fabrication technology and discuss the evolution of architecture in the light of material as well as integration for signal refinement and wireless data transmission.

**Fig. 1:**
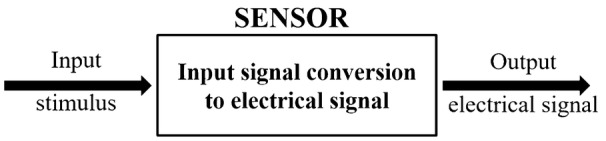
Basic structure of a generic sensor consisting of the conversion circuitry that transforms the input energy to electrical signal

A sensor is defined as a device which transforms the applied form of energy into a measurable electrical quantity, which is an essential feature of the device. Though sensor functions as a transformation of input energy to electrical form, it is often understood for a transducer wherein the transformation can take form of any kind of energy, either electrical or non-electrical. However, a sensor converts the input stimulus only to an electrical form. A generic sensor can be represented as in Fig. 1.

Sensors are classified based on different categories. In a broad perspective these are classified as direct and complex; active and passive; absolute and relative. A direct sensor is one which converts the stimulus into an electrical signal or modifies the electrical signal by using an appropriate physical effect. The complex sensors would require one or more transducers before a direct sensor is implemented to generate an output electrical signal. Active sensors on the other hand need an excitation signal in the form of an external power. This external signal is modified by the sensor to produce the output signal. These are also termed parametric as their inherent properties are modified in response to the applied external effect. A passive sensor on the other hand needs no powering circuit and generates an output signal from the applied input stimulus. An absolute sensor provides a response irrespective of measurement conditions. For example, a thermistor gives an electrical resistance in the absolute Kelvin scale at a particular temperature. A relative sensor has a particular baseline calibration relative to which it responds to applied stimulus. A pressure sensor operated at atmospheric pressure is a relative sensor that gives an electrical output at pressures higher than the atmospheric conditions. A sensor is thus devised for a particular application and operating conditions falling in one of the classifications applicable [1].

Sensing action in any device follows an underlying principle by virtue of the material or the architecture of the device. A feasible mechanism of device most commonly used has either a piezoelectric, resistive or capacitive behavior. These have a sensing methodology for each of the device architecture for the sensing element in them. These sensing elements ensure sufficient resolution and sensitivity when paired with the appropriate electrode. The device development has seen several forms for these sensing elements that mark the different milestones in their overall development. Thus, the evolution of sensors has been a time seen procurement. These began when human needs emanated for remote and quick unmanned control and monitoring of processes. The facilities tempted mankind to nurture new devices with innovation along with simple and easy methods for the fabrication cost and time constraints. The early stage sensors constituted of a simple diaphragm and electrodes with well-defined sensing regimes in the touch region of sensing [2–5]. Later developments were incorporated in sensing other analytes ranging from chemical sensors to physical vibration. These application specific evolutions have seen several advancements both at the material level as well as the device configuration and architecture. Later advancements came in the form of textile based sensors incorporating a conducting element into the yarns or fibers. A detailed insight into the progress confronted in the development of sensors is detailed in the sections that follow based on the advancement in the light of materials and architecture. Several innovative designs have been implemented for the ease of fabrication or the cost and time efficacy achieved. Any new aspect in this regard has been a novel approach and has been abridged in the sections below.

## Primitive to modern sensors: an abridged review

**Fig. 2:**
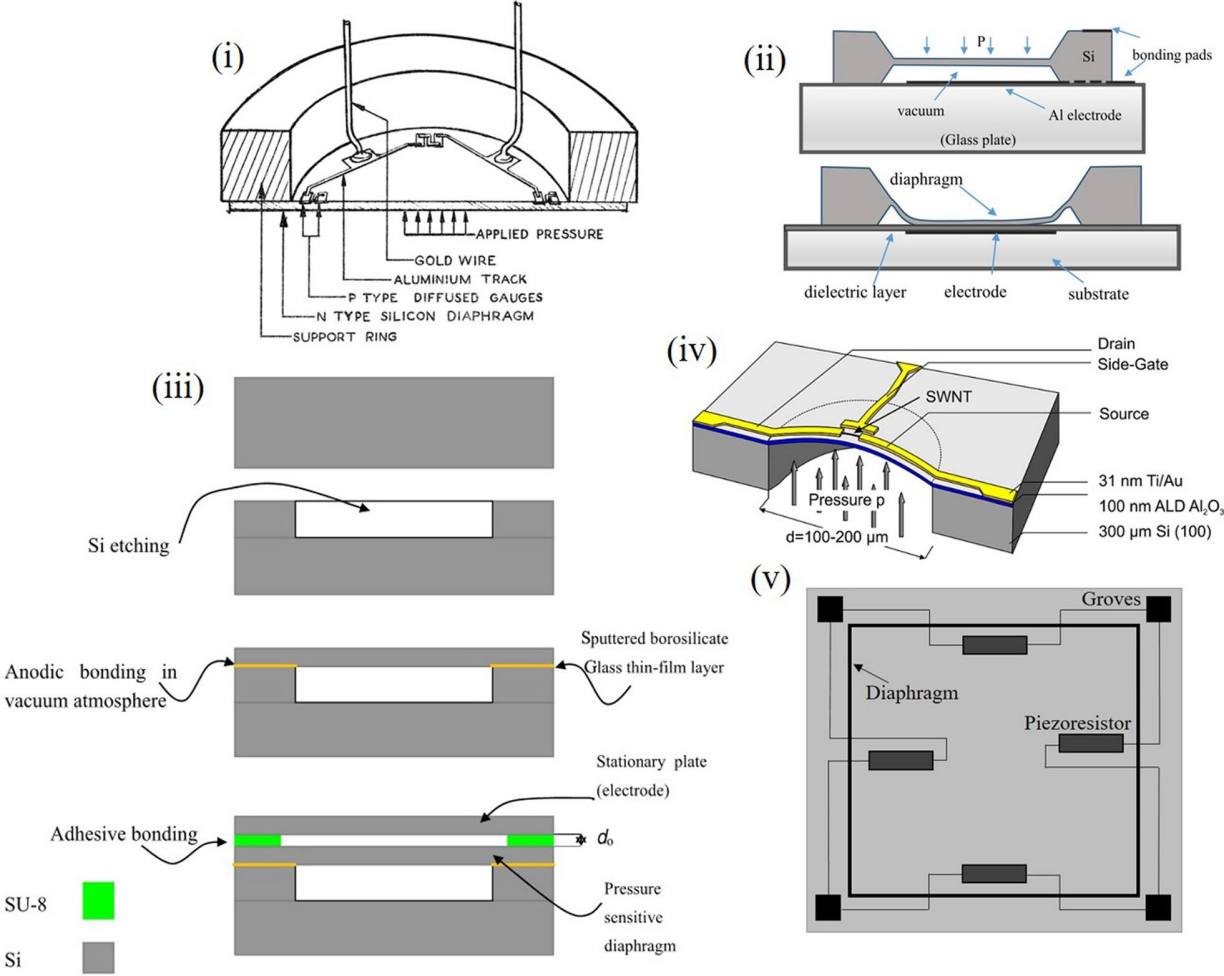
Early silicon based pressure sensor fabricated with MEMS (i) Complete Si wafer based crude design (Reproduced with permission from [7] *Copyright 2007, Emerald Publishing Limited*) (ii) Etched Si diaphragm (Reproduced with permission from [8] *Copyright 2016, Copernicus Publications*) (iii) SU-8 supported diaphragm (Reproduced with permission from [8] *Copyright 2016, Copernicus Publications*) (iv) Carbon Nanotube (CNT) strain gauge based peizoresistive sensor diaphragm (Reproduced with permission from [182] *Copyright 2006, American Chemical Society*) (v) S shaped diaphragm with piezoresistive material at the groves for improved sensitivity

Sensing was realized an essential entity when direct interpretation of non-quantifiable form of energy became indispensable. It became essential for obtaining this form of energy into an easily and directly interpretable form leading to the development of sensing devices. Sensor fabrication began with the advent of the fledgling semiconductor industry when the lithography process began in the early 1950s with possible advancements for the diaphragm model. These were fabricated using the conventional lithography tools that existed in the silicon industry and etching of silicon was well established to obtain micro engineered silicon wafers. The early sensors had a basic suspended diaphragm between two electrodes in its capacitive configuration. Any mere change of the external stimulus could provide a change in the output parameter measured; the capacitance of the device. This diaphragm fabrication marked several advancements in the design which began with the micro-electro-mechanical system (MEMS) based devices. These became outdated for the fabrication efforts and saturation attained for the maximum utility that could be exploited. The early stage sensors were focused in implementing unmanned machining for checking the dimensionality, quality and orientation of work tools in machines, its monitoring and checking wear and breakage using vibration sensor [4, 6–8]. Figure 2 shows typical MEMS based pressure sensor devising a silicon wafer patterned for a diaphragm and electrode using lithography and doping based etching technique.

A typical MEMS based strain/pressure sensor response is identified with four regions over a wide pressure range namely normal, transition, linear and saturation [9]. The linear region forms the useful operational region for the sensor and defines its sensing performance. This classification was formed based on a touch mode sensing action wherein a touch based external pressure applied to the diaphragm is transformed into capacitive output. In the normal operation mode, diaphragm does not touch the substrate electrode. The output capacitance is nonlinear with inverse relationship with gap which is a function of pressure. The linear region commences with mechanical contact of diaphragm with the substrate. Here, the major component of capacitance is the touched area where the effective gap is thickness of the thin insulator layer on the substrate holder. Because of the small thickness and large dielectric constant of the insulation layer, the areal capacitance is much larger than that of the untouched area. Once the diaphragm touches the substrate with increase in applied pressure, sensor capacitance is mainly determined by the capacitance of touched area instead of the normal operation portion area having a nearly linear response. Touch mode operation has good linearity and sensitivity with the substrate giving good support to the diaphragm after touch with large overload protection [2]. These were confined to the electronics industry alone for the lithography techniques involved in them. However they failed in fields of gas sensing, biomedical sensing for their competitive counterparts for efficient performance for incurred fabrication cost and time.

Performance enhancements have been studied in terms of dimension and shape of the diaphragm for the desired region of operation for attaining linear response. The varied shapes of square, rectangle and circular have been studied with scope of sensitivity improvement. However, any scope of advancement to the silicon based devices lacked the flexibility and conformity to curved surfaces leaving behind little scope for progress [6, 10–15]. These were thus confined to large pressure applications due to less scope of capacitance that could be obtained from the rigid architecture. MEMS paved way to NEMS (Nano Electro Mechanical systems) as a step towards miniaturization. However the performance could not be better than conventional devices. It thus was indispensable for other materials and architecture to grow for satisfying the emerging needs of time.

With the advent of thin membrane fabrication along with elastomer materials in the early 1970 s and their in-depth study of surface energy and adhesion with rigid surfaces, a drift towards utilizing elastic materials for conformal sensing bloomed [16, 17]. Elastomer solids with their versatile Young’s modulus tuning and application specific soft lithography enabled them for speedy and easy design and device developments. These have also been developed for composite materials for highly effective pressure and strain sensing for their passive, submissive and inert nature to most chemicals further adds advantages of imparting large number of options for a myriad of applications. Composites of many forms beginning from coated conducting elastomers to embedded composites have been studied utilizing various compatible conducting moieties either modifying the base elastomer or itself to bind with the matrix elements for enabling stretchability has enhanced possibilities in the field sensing and provided a new front for progress. The following sections discuss these in detail with an abridged review on the innovative advancements in the design and development of sensors from both a material as well as architecture perspective.

## Choice of elastomer material for sensor

All entities in nature have their intrinsic properties that help them remain unique. Like so, elastomers have a typical mechanical property by virtue of which they are capable of accommodating the stress induced or generated in them giving a linear region in the stress–strain behavior. Though elastomer have recently gained importance in sensing regiment, their structural and adhesive nature with rigid surfaces have been studied extensively since their inception in the early 1970s [16]. Elastomers are now commercially available in the form of Ecoflex [18], polydimethylsiloxane (PDMS) [19–21], poly(styrene–butadiene–styrene) (SBS) [22–24], polyurethane (PU) foam [25], Silbione [26] and Dragon skin [27]; each of which possess a characteristic range of mechanical properties. A large range of Young’s modulus is thus available to us for an apt selection of material and range of operation. Of these PDMS is widely used and provides the widest window of operational variability in Young’s modulus that can be customized and tuned with mere temperature and time variances [28, 29]. This keeps it aloof of all other available elastomers. However, other elastomers are also being explored for their unique properties like better elasticity compared to PDMS for better sensitivity. Each of the available materials is modified in their appearance and morphology by varied processes using one or more agents for specific functionalities. Each of the specific processing technique provides performance by virtue of the physical properties of the form. However, devices with elastomer in their pristine as well as composite form impart in them electrical properties which are measured during the transformation of energy from any form to electrical form in the sensing action of the sensor. These varied forms are elaborated in the form of evolution of innovative device architecture in the sections below.

## Evolution of sensor device architecture

Sensors come in manifold applications which convert the physical form of energy into electrical forms for easy measurement and interpretation. Several forms of these exist like pressure/strain sensors, temperature, humidity, sweat etc. In a pressure/strain sensor, the change in pressure/strain sensed by the device is quantified from its piezoelectric response. These pressure sensors are easily realized in one of three configurations namely, the piezocapacitive, piezoresistive and the capacitive model. Varied configurations have their pros and cons. However, the capacitive configuration finds an upper hand. These have a better drift response, stability, lower power consumption, less prone to misalignment errors which are critical in piezoresistive sensors, ease of fabrication and low cost fabrication possibilities and many more [3]. The capacitive model is inferred to be highly economic with little dependence on ambience and provides highly repeatable response [1, 30]. A change in the dielectric thickness is accepted for change in capacitance delivered in response to the applied mechanical stimulus [30]. Pressure sensors are defined by their sensitivity delivered in a pressure range giving them the low or high pressure sensor nomenclature. A few devices provide good sensitivity in the low pressure region; while others provide in the high pressure regime. The processing and fabrication are thus adopted for a target application. Low pressure ranges are considered up to 10 kPa; up to 250 kPa are considered as medium pressure and above 250 kPa as high pressure range. Sensors for each specific category have been achieved with sufficient sensitivity by adopting different configurations and materials which forms the integral part of this section.

### Bumpy designs

PDMS silicone material by virtue of its rapid prototyping soft lithography molding capability can be processed easily and quickly within its window of operation [21, 31]. The conventional procedure has a mold patterned using photolithography on silicon substrate with pattern defined by SU-8 negative photoresist for the required design dimensions. The basic sensing mechanism in these bumpy devices is the squeezing out of an air gap increasing the capacitance. These devices are fabricated both using MEMS based techniques as well as rapid prototyping. These also exhibit good performance for the range of operation and sensitivity ranging in the touch mode sensing to plantar pressure sensing [32–37].

**Fig. 3:**
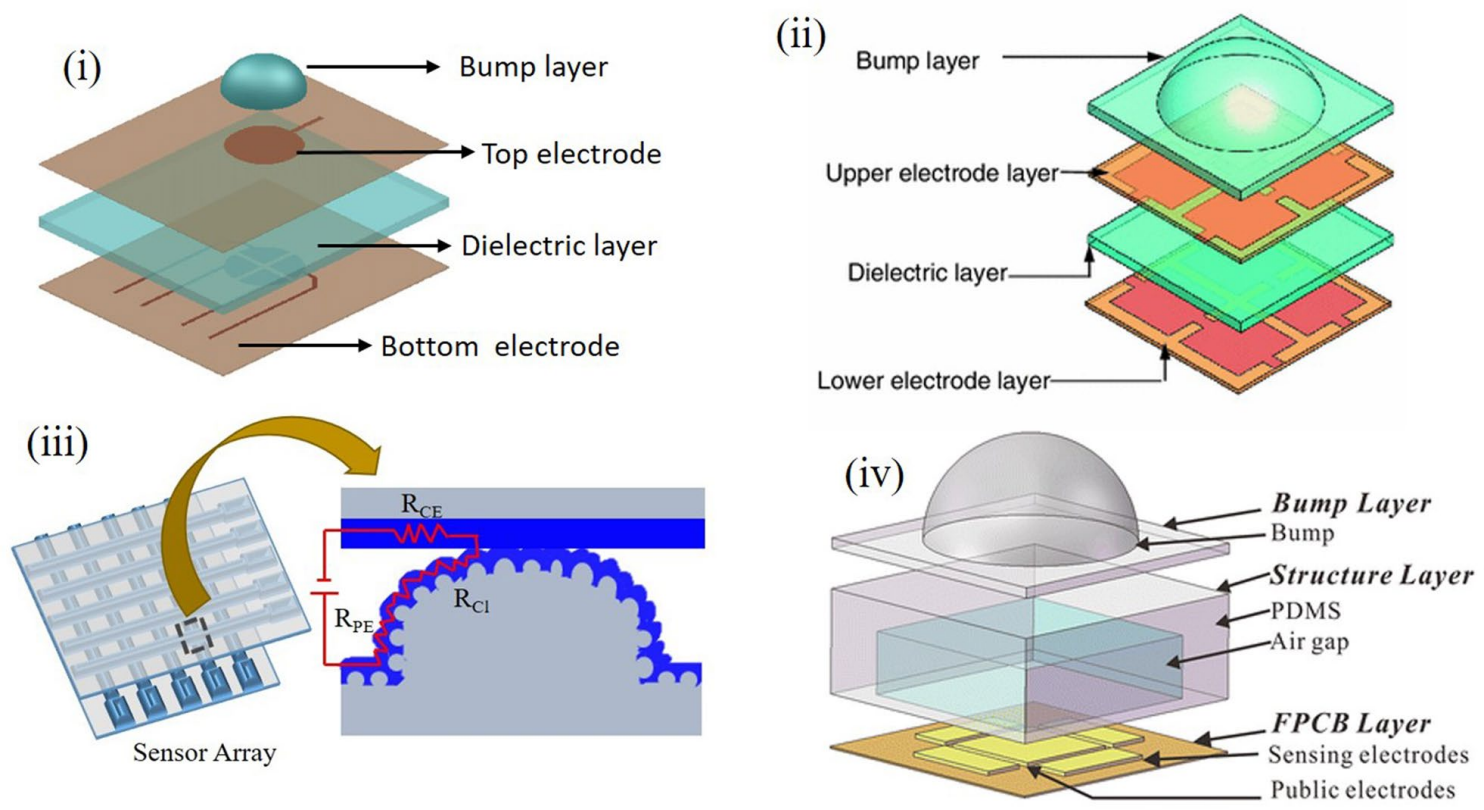
Bumpy design PDMS sensor with (i) Plain top electrode (Reproduced with permission from [33] *Copyright 2012, Elsevier*) (ii) Divided top electrode configuration (Reproduced by permission from [34] *Copyright 2013, Springer*) (iii) Poly(3,4-ethylenedioxythiophene) poly styrene sulphonate (PEDOT:PSS) enhanced conductivity. (iv) Air gap model with PDMS bump (Reproduced by permission from [183] *Copyright 2016, Springer*)

Basic sensor architecture based on the bumpy layer are shown in Fig. 3. It has a layered fabrication process with each layer requiring one or more lithography and alignment steps with subsequent metal deposition. The bump layer was implemented to provide an even distribution of force for compressing the PDMS dielectric layer giving a capacitance change. Both normal and shear forces were illustrated using the bump design structure by Cheng et al. where they used bump layer along with entrapped air gap embedded within the bump and substrate elastomer for both strain and shear pressure measurements [38]. Another approach in the same light was illustrated by Lee et al. with an improved design of suspended air gap for better processing and sensing. This has the side walls of gap removed giving a suspended air gap enabling three dimensional (3D) shear force sensing [35]. These were further implemented widely in plantar pressure measurements using the combination of air gap embedded device and divided electrode design. Plantar pressure sensors were initially undertaken by the MEMS based technology; however, their bulky design limited its pixelization causing the drift in their architectures. Bumpy layer facilitates even pressure distribution over the entire pixel giving uniform response rather localized changes at the material level [34]. Further, Xi et al. introduced a conductive layer of rubber between the bump and substrate and used the divided electrode design giving a resistive sensor rather the conventional capacitive mechanism. Here, when the bump is pressed, the thickness varies and resistance of the conductive rubber varies with respect to the applied pressure [33, 37].The advantage of bump layer over planar design was the out of plane shear and three axis strain measurements [35, 38, 39]. Liang et al. sandwiched an additional microstructured layer of PDMS beneath the bump and electrode layers for improved sensitivity. They patterned a truncated pyramidal structure for the PDMS by etching techniques. This truncated pyramidal structure enables the polyethylene terephthalate (PET) electrode layer to regain its form after deformation giving faster response times compared to pointed pyramidal structures [40]. Fang et al. improved the resistive behavior by a conductive coating of PEDOT:PSS over the microstructures giving a resistive sensor from the bump layer design [37]. Rather getting a single device fabricated, an array for large area pixelated sensing has also been realized with these bumpy designs for their lithography based approach enabling large area patterning and batch processing. These have also been used in sensing the critical points in plantar pressure measurement at large scale. These sensors were used in a broad range of pressure sensing from a few Pa to several kPa by tuning the PDMS curing agent weight ratio and bump designs.

### Microstructured elastomer

**Fig. 4:**
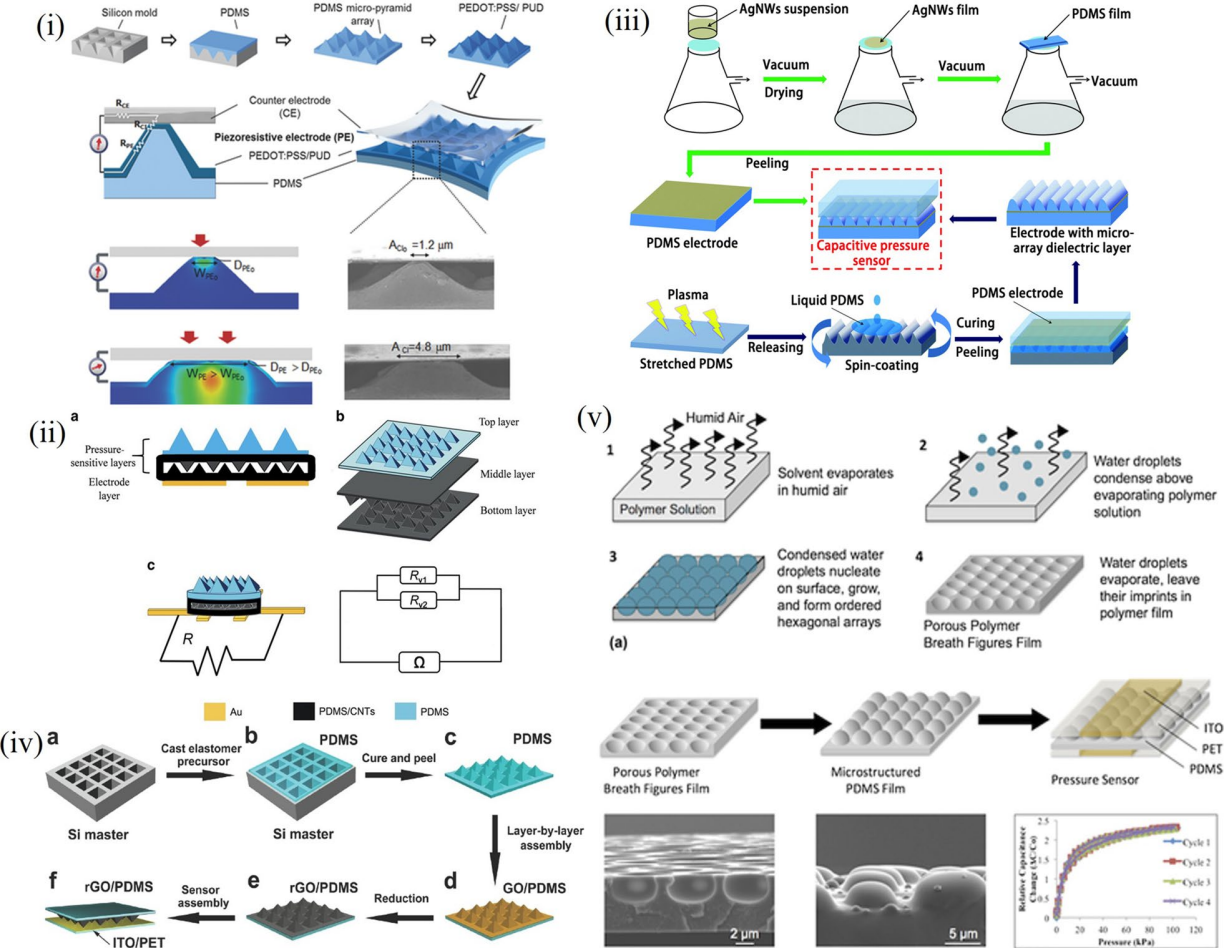
Patterned elastomer based strain/pressure sensor with varied approaches (i) Conducting polymer induced enhanced conductivity(Reproduced by permission from [49] *Copyright 2014, Wiley*) (ii) rGO/PDMS patterned (Reproduced by permission from [58] *Copyright 2016, Springer*) (iii) Plasma induced electrode defined on wrinkled PDMS (Reproduced by permission from [50] *Copyright 2018, Royal Society of Chemistry*) (iv) Compound elastomer pattern with complimentary rGO/PDMS films (Reproduced by permission from [47] *Copyright 2014, Wiley*) (v) Patterned PDMS with breath figures (Reproduced by permission from [51] *Copyright 2014, IOP science*)

Elastomers by virtue of their wide processing window and soft lithography are best suited for sensors added to their surface conformity. Initial reports on soft lithography was studied for the varied possibilities of patterning elastomer [41–45]. PDMS by virtue of their easy fabrication was preferred candidate for the purpose of obtaining patterned elastomer. The approach here revolves around obtaining a silicon mold of desired microstructure via conventional photolithography and chemical etching. The microstructure shape and aspect ratio is optimized for the viscous nature of elastomer; PDMS which is widely used. Initially only capacitive mechanism was implemented which were later extended to resistive approach as well. Figure 4 elaborates these sensors. Mannsfeld et al. elaborated this approach on large scale sensing by using varied shapes for the microstructure. Pyramidal and line structures were made for the elastomer film; pyramidal structure by virtue of higher aspect ratio provides higher sensitivity for the greater change in capacitance for applied pressure for larger voids in the film. This shape influenced capacitance by voids present in between the microstructures providing feasibility of sensitivity control with additional control of elastomer mechanical properties with curing temperature and curing agent to base weight ratio [46]. The sensor sensitivity arises from reduction in distance between the electrode plates and is further enhanced by the effective dielectric constant. The saturation regime sensitivity drops as the elastic resistance increases with increasing compression. This approach also enables tuning of sensor parameter by shape of the microstructure. Further, Zhu et al. illustrated thin films of these patterned elastomers coated with graphene of thickness in a few micrometers. Similar approach of template molding was carried out to procure patterns over the elastomer film. Graphene oxide was then deposited over these by a layer by layer mechanism and reduced to form conducting layer of reduced graphene oxide (rGO). This piezoresistive film is then sandwiched between flexible conducting sheets to form a complete sensing device. This allows for large area pixelated sensing forming an array from the mold patterning approach. This also allows for batch processing once the mold is prepared accurately for precise replication of microstructure shape and size [47]. Chou et al. implemented partial removal of conducting graphene from the pyramidal surface to increase the base resistance value and to impart better sensitivity to the sensor. The graphene layer was uniformly coated over the pyramidal structure and it was removed from the top surface alone using a kapton tape [48]. Although these possess good repeatability and performance, a few parameters like multilayer fabrication and time and expense in mask development inhibits the use of microfabication. Further, the visco elastic property of PDMS limits the aspect ratio in 0.2 to 2 for efficient repeatable patterns. Repeated use of single mask causes elastomer debris to damage the accuracy of mold. A sacrificial layer or treatment of the carrying substrate is thus required to prevent adhesion and smooth peeling off of cured PDMS film for evenly patterned surfaces. However, patterning the elastomer surface is not limited using a rigid mold but also upon other surface exposure techniques as well which render the surface a periodic pattern at the microscale [44, 46, 49–56]. Miller et al. elaborated breathe figure method for obtaining uniform pattern on PDMS using water droplets. They refer to the organized arrays of water droplets that form when humid air comes in contact with cold solid or liquid surfaces.

Water droplets from a flow of humid air condense above the surface of a polymer solution, cooled by the evaporating solvent. This results in a highly ordered, monodisperse array of water droplets that leave their imprints in the form of microscale honeycomb arranged cavities on the polymer film after the solvent and water droplets evaporate. This forms a highly cost effective and simple approach of patterning elastomer compared to the conventional lithography processes. Initially a porous film template is fabricated in a humidity controlled chamber with polystyrene balls dispersed in chloroform for uniform breathe figures. Upon this porous film PDMS is patterned via soft lithography [51]. While patterning the dielectric layer was common trend, Cui et al. described a sensor with solid dielectric layer and patterned electrodes. A V-array shape was chosen for the electrode with a double etching and lithography process to replicate the V shape rather its negative. The pattern was transferred via nano-imprint lithography prior to PDMS molding. Further, silver embedded PDMS electrodes were used to form a complete sensor device [57]. Wang et al. used a multi patterned layer architecture to extend the range of operation and better sensitivity. Three layers of pyramidal PDMS were employed to form the resistive sensor. The top layer is made to act as the bump layer for better distribution and concentration of applied stress and thus improve the sensitivity. The middle layers made of PDMS/Multiwall CNT (MWCNT) are glued at the ends forming parallel resistors giving an increased resistance with applied pressure. This approach with added flexible electrodes provides large area pixelated sensing platform with least crosstalk as the upper pyramidal layer alone defines the impinging pressure [58]. Li et al. extended this approach by defining intrinsic and latent connections of the pyramidal tip with patterned electrode for different range of applied pressures. During the intrinsic connection, current flows through the deformed MWCNT coated pyramidal PDMS layer and electrode, as parallel resistors as the distance between them decreases under applied pressure; the latent connection is formed when the pyramidal tips contact the electrode [54]. Other ways of obtaining microstructures over PDMS were explored with aim of bringing down the cost and time of fabrication. In this light, Ma et al. proposed plasma mediated wrinkling of PDMS sheet for use as dielectric and conducting electrode when coated with silver nanoparticles. Plasma also enables uniform coating of the conducting solution giving a uniform conductivity [50]. The patterning approach was further extended to other polymers and composites which provide a synergetic function of both a power generator as well as sensor. Lee et al. patterned Poly (vinylidene fluoride-co-trifluoroethylene) (P(VDF-TrFe)) films to obtain the synergistic sensing and power requirements for making it a standalone device [59]. Choice of Polyvinylene diflouride (PVDF) becomes essential over other available piezoelectric materials for its easy synthesis, transparent nature and mechanical sturdiness. Other forms explored in this venture were in the form of a mechanical distortion used to produce required power to stimulate the sensor output, which can also be in visual form of a color change achieved by incorporating electrochromic materials [48, 60–64]. Chou et al. implemented a layered design incorporating pigmented cells for the chromic response and CNT coated pyramidal PDMS layers for the pressure sensitive layers [48]. They elaborated on the low resistance of pyramidal structure and integration of an electro chromic device for color display of applied pressure. Recent trends have evolved towards making the sensor device self-powered taking a step further towards wireless sensing. Patterned films have provided good performance with regard to the self-powered devices for the greater deformation possible compared to unstructured thin films. Fan et al. showed the effective use of pyramidal PDMS films as both sensors as well as tribo generator simultaneously. The pyramidal patterns were replicated onto PDMS by conventional lithography process on rigid silicon wafer using a silane solution for maintaining accuracy for repeated use of the mold. The sensor device fabricated effectively showed self-powered sensing for the larger output by virtue of the micro patterns formed on the elastomer film [60]. These patterned films of PDMS were also functionalized by metallic coatings. Dhakar et al. exploited the conducting nature of epidermis for charge generation and separation with gold coated patterned PDMS layers for a generator cum sensor device [62]. The pyramid pattern was reported to give maximum sensitivity both as a sensor as well as nano-generator driven sensor device for the greater void area present between the electrodes giving a better scope of deformation. However, the demerits of microfabrication still persist in these approaches. Apart from microstructured elastomer being used in pressure sensors, these are being extended towards realizing electronic skin. Park et al. demonstrated replicating finger print like patterns onto PDMS along with ferroelectric composite layers for effective sensing of static/dynamic pressures and temperature [64]. Each microstructure has been thus derived to serve a distinct application in the most effective way possible and improve the efficiency of the sensor. These microstructured elastomer films help implement a self-powered sensor with dual function of generating power from the applied pressure. As the patterns could be replicated uniformly on any number of molds, batch processing and thus array fabrication could be feasible with this approach that has led to large area sensing and critical point sensing as well. The homogeneity that could also be maintained with the well-established processing methodology also enables this method to be used as a standard protocol for large area sensing applications.

### Elastomer composites

**Fig. 5:**
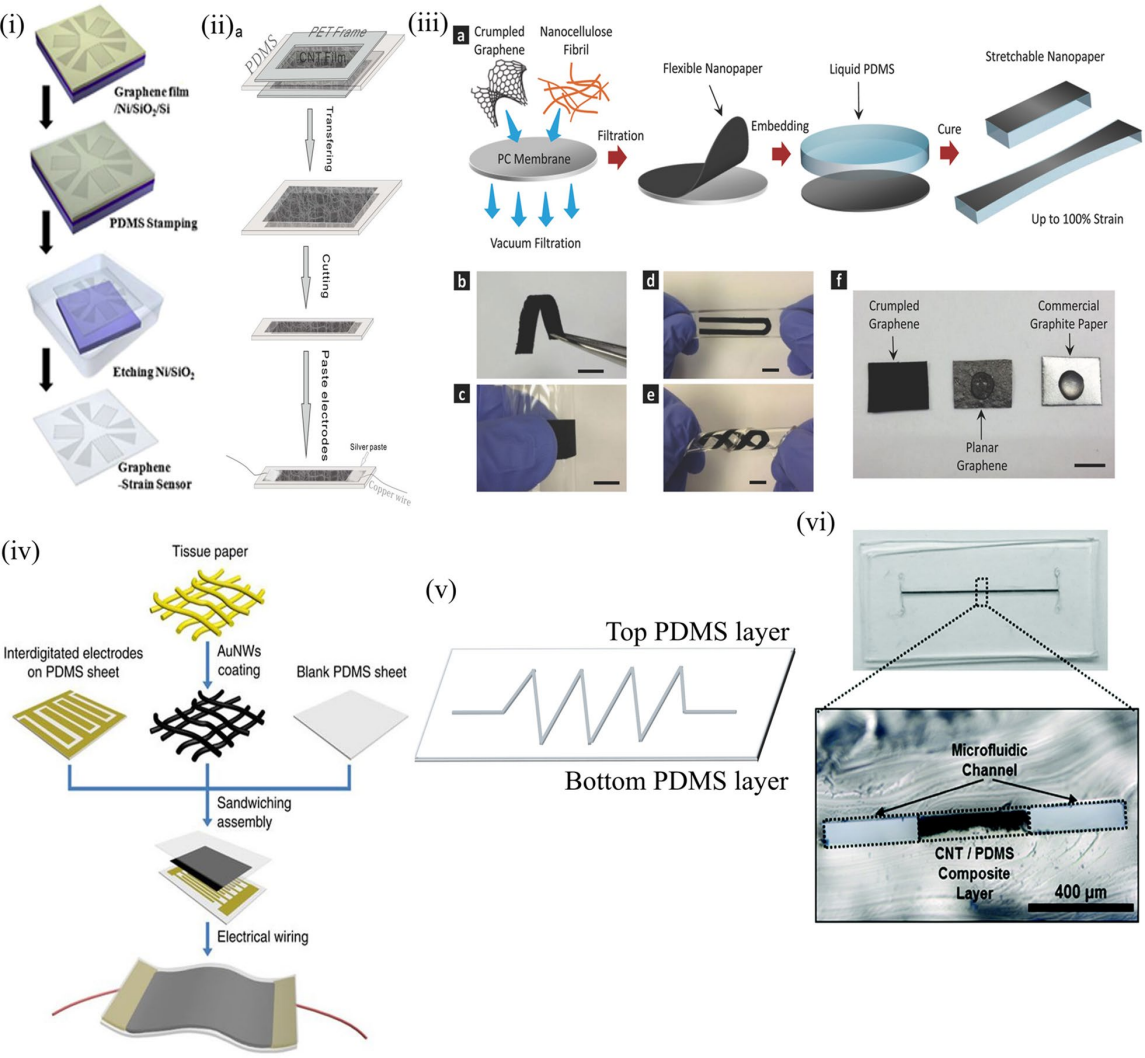
Sensors obtained from composites of conductive fillers in (i) Graphene film deposition (Reproduced by permission from [184] *Copyright 2013, Elsevier*) (ii) CNT film between PDMS slabs (Reproduced by permission from [135] *Copyright 2013, Nature*) (iii) Graphene infiltrated cellulose paper (Reproduced by permission from [185] *Copyright 2014, Wiley*) (iv) Silver nanowire (NW) coated on tissue paper with interdigitated electrodes on PDMS sheet (Reproduced by permission from [186] *Copyright 2014, Nature*) (v) Microstructure sandwich form. (vi) Composite encapsulated in a microfluidic channel (Reproduced by permission from [78] *Copyright 2017, Royal Society of Chemistry*)

Elastomers are inherently insulators and were thus used as dielectric material in many of the capacitive sensors. However, their solvent compatibility [65] and inert nature to most carbon based and other metallic materials makes them ideal for providing conductive nature retaining their elastic behavior [66, 67]. Their compatibility with most available conductive fillers allows them to be used as the foundation layer of all sensing elements in both capacitive and resistive modes. This configuration has an initial process of making the composite paste. It is then applied onto conductive substrates using one or more of existing methods. The uniformity of the film depends largely on the composition of the conductive composite paste which imparts desirable performance to the sensor. A solution method is followed with initial dispersion of carbon based nanomaterials in the form of graphene, carbon nanotube either single wall or multi wall with single wall providing better conductivity, or carbon black in a mutually suitable solvent for complete mixing of the resin and filler components. With evaporation of the solvent the curing agent is added giving a cured conductive film with piezoresistive electromechanical response [27, 54, 67–75]. The composite is then made into molds of desired shape and size to obtain individual devices or as an array for a complete sensing patch. Initial composite based sensors were fabricated using a paste of piezoelectric material with solvent for printability [76]. Though employed as a vibration sensor that was well established in the MEMS technology, this marked the beginning of the use of composites in sensors. Carbon based fillers were studied initially for the compatible common solvents for both PDMS and the filler. Figure 5 below illustrates the composites based sensors. Dusek et al. employed carbon black in PDMS and implemented a hydrodynamic sensor as well as strain sensor modifying them into porous sheet using sugar templates [71]. Jung et al. exploited the composite structure by using five directional sensors for discriminating multidirectional forces based on the resistance changes evolved in each element for a directional force. This architecture involves a walled design along with a central locked composite structure for enabling both normal pressure sensing as well as shear force sensing [77]. Apart from carbon based materials, other conducting metallic nanomaterials and piezoelectric materials are also used for forming composites. These composite mixtures can also be patterned by photolithography techniques. You et al. patterned silver nanowires using photolithography in a tandem pattern and were embedded with kapton sheet aiding its flexibility [69].

For every filler material used, the percolation limit defines the sensing capability of the device. A trade-off between conductivity and stretchability is observed for higher weight percent of filler added to the elastomer which limits the range of operation of the device. This limitation was rectified by used of patterned composites and other more conducting fillers like silver nanowires, MXenes, ionic liquid gel, zinc oxide nanowires, liquid crystals etc. and also by forming a microfluidic channel of the composite. The device fabrication in these has PDMS that acts as both an electrode substrate and as substrate for the nanomaterials. The conducting entity is then made a fine thin film and sandwiched between PDMS films for supporting the stretchability of the rigid entity. ZnO nanowires are vertically grown over the PDMS substrate to form two parallel surfaces that form a piezoresistive device. Their bend and stretch provides corresponding electrical response giving a pressure or strain sensing. For other nanomaterials, a similar electromechanical response is derived from a change from their pristine position [78–87]. These composite materials were also employed for simultaneous energy generation by employing a tapping action for the charge separation and recombination to allow for charge transfer towards realizing self-powered sensors. Rasel et al. employed a spring design for facilitating the tapping action for efficient charge storage and separation. MWCNT doped PDMS enabled tuning of resistance of the sensing layer giving it a dual function of resistive sensing and charge storage [61, 63]. These composites have a systematic fabrication procedure that can be carried out at open ambience and are thus facile for area and dimension scalable sensing platforms. With the advent of 3D printing, the outer encapsulation for these became more facile enabling customized casing and device protection.

### Elastomer sponge based pressure sensor devices

**Fig. 6:**
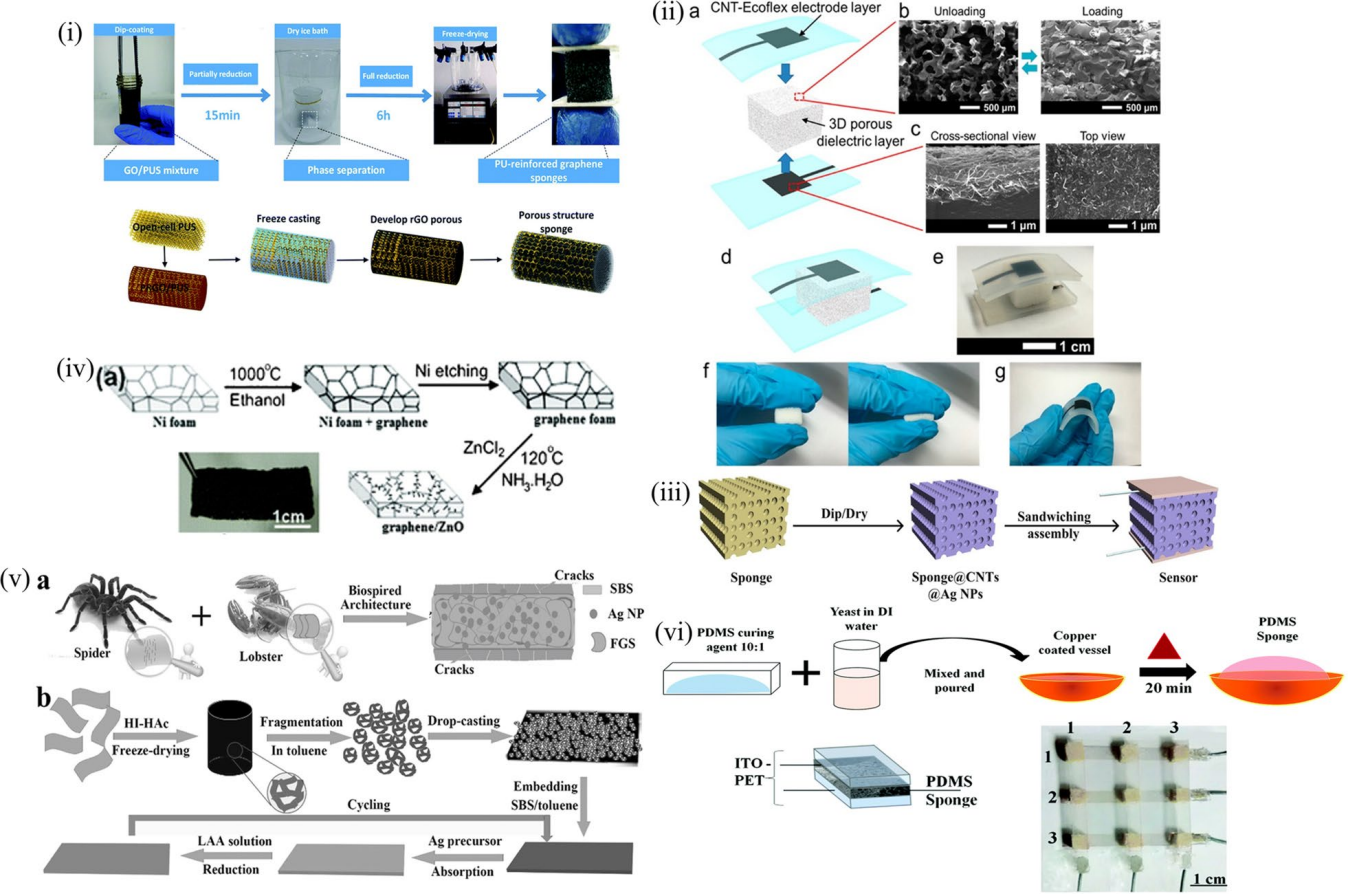
Porous elastomer based sensor (i) PU sponge based porous grapheme structure (Reproduced by permission from [91] *Copyright 2017, Royal Society of Chemistry*) (ii) Elastomer sponge made from sugar cube template for capacitive sensor (Reproduced by permission from [18] *Copyright 2016, American Chemical Society*) (iii) Commercial sponge made resistive with CNT-Ag NP (Reproduced by permission from [82] *Copyright 2016, American Chemical Society*) (iv) Metal mesh assisted resistive sponge (Reproduced by permission from [121] *Copyright 2012, Royal Society of Chemistry*) (v) Microstructure crack assisted enhanced sensing from bio-inspired architecture (Reproduced by permission from [86]. *Copyright 2017, Wiley*) (vi) Yeast autolysis assisted porous PDMS for capacitive sensing (Reproduced by permission from [19] *Copyright 2018, Royal Society of Chemistry*)

Elastomers as seen in previous sections have been modified for implementing pressure sensors. Another architecture that has been in focus since late 90’s but was extended towards obtaining porous elastomers was a morphology varied approach wherein the elastomer is made porous and used as the dielectric in capacitive sensors. These were later implemented as resistive when the hydrophobicity of it was overcome by either plasma treatment or by a surfactant. The porous structure enables the applied pressure/strain to be accommodated within the strands or pore walls giving a linear relationship for the stress–strain curve. Pores occur either in the open pore or the closed pore forms. Open cell form deformation was studied in detail by Moore et al. [88]. The linear region is observed for ~ 5% strain. This region is associated with bending of the cell ribs though earlier it was advocated by models as a rib extension. Rib bending gives rise to a modulus which increases as square of the relative density in the linear region. At higher strain, buckling of these ribs give rise to plateau region followed by densification by contact of these ribs and is interpreted in the context of stability. Elastomer porous sponges as well as readily available sponges are implemented for low cost large scale sensor array applications [82, 89–97]. Figure 6 below elaborates the approaches. Polymers were initially made porous for their varied applications [98]. These can be of either physical or chemical nature. Physical methods include obtaining the final porous template by a lithography step [99–108] or adding a porogens material prior to PDMS curing and leaching away the porogens [18, 30, 90, 95–97, 108, 109]. Chemical route essentially involves a chemical reaction between the solvent and porogens leaving pores in intact PDMS resin [15, 19, 110–114]. Juchniewicz et al. used distilled water as porogen for obtaining porous PDMS along the walls of a microfluidic channel by pumping gas after the mixture is injected into the channel. They observed that for different sequences of adding the components, only homogeneity of the mixture was critical for the nature and size of pores formed. The curing temperature was also found to affect the pore features [115]. Here when plain water was used as the porogen, Ou et al. used polystyrene beads in toluene to dissolve both the PDMS and polystyrene and the solvent was evaporated leaving behind the porous elastomer. These were employed in protein separation using isoelectric focusing mechanism [116]. Qi et al. used a hard template of citric acid monohydrate particles and ethanol as solvent for obtaining composite sponge of CNT-PDMS for fabrication of both a single cell and arrayed configuration with high gauge factor [113]. Wei et al. on the other hand used photoresist posts to fabricate porous PDMS [103]. Choi et al. introduced the use of sugar cube templates for obtaining PDMS sponges by leaching away the sugar particles after complete curing of elastomer.

Sugar cube provides shape and dimensions to the final sponge obtained giving pore features of dimensions of the voids in it [117]. Overall, a template assisted method forms the basis of obtaining porous features in the elastomer. Thus the feature sizes are determined by the template. Hence with proper control of the template features, the final porous elastomer also could be manipulated. A step further was made by Murray et al. using ammonium hydrogen carbonate in PDMS matrix and curing it for simultaneous curing of the elastomer and porogen removal [118]. Another milestone in obtaining porous elastomer was using photolithography using a phase mask. The phase mask has periodic arrangement of pillars of PDMS forming conformal contact with the photoresist. The periodicity defines the 3D structure of the porous PDMS obtained in the resist upon UV exposure. Nam et al. provided a density gradient on the nanoporous features using simple photolithography [119]. Jeong et al. used steam etching to make localized porous PDMS and developed stretchable conductors [120]. These are a few methods for obtaining porous PDMS for their versatile applications. A two way method of obtaining resistive sponge has been illustrated until. One route is obtaining the porous elastomer and then incorporating the conductive filler in it by a dip-dry method. Another is by obtaining the porous conductive entity and then infiltrating them with PDMS and curing. In both cases sensing is enabled from the conducting strands with former more preferred for the facile nature of approach. The latter is found to have limited repeatability for the higher hysteresis in the load-unload cycle. These porous elastomers provide a strain sensitive effective dielectric constant (for capacitive) and resistance (for resistive) variation resulting in a corresponding change in its output giving the sensing action to the device. The compressive stimulus pushes the pore strands together giving a modified effective dielectric constant thus modifying the device output capacitance. For the resistive sensor, the compression causes the conducting strands to shorten the conducting path length reducing the effective path length reducing the resistance of the device. The microstructure achieved from the physical and chemical routes enables uniform pores to form dense pore strands for efficient sensing of wide range of pressures. For better sensitivity, the sponge is kept under compression for a microstructure modification for long hours giving a higher sensitivity. The three dimensional structure enables accommodation of applied strain/pressure giving a lower Young’s modulus compared to the bulk material. Kwon et al. employed a capacitive sensor made from PDMS sponge with grapheme coated ecoflex sheets as electrode for maximizing the device sensitivity. The sensing action is reported to have taken shape from both the porous nature of PDMS as well as the conformal sensing of ecoflex [18]. Zhang et al. implemented a resistive sponge by incorporating CNT Ag nanoparticles hybrid for enhancing their conductivity for better sensing [82]. Yao et al. exploited the microstructure fracture that can be incorporated with prolonged compression of PU sponge dipped in graphene solution for achieving higher sensitivity. The idea implemented was inducing deliberate fractures in the sponge microstructure leading to better contact resistance upon an applied external pressure [92]. Wu et al. used a layer by layer deposition of carbon black over commercially available PU sponge for getting a better homogeneity of the conducting network. The charge difference in the PU and carbon black ensemble enabled the uniform coating of it over the strands giving better adhesion and long term reliability of the device. These were also implemented for array application for ease of fabrication and repeatable electrical response of the standard approach [93]. This regime became stagnant with all feasible approaches for modifying the inner porous nature of otherwise solid elastomer that helps define a net change in the resistive path or the effective dielectric constant that define the electrical parameter with a single step method void for the same. This gap was filled with the simultaneous curing and autolysis of yeast introduced by Chithra et al. with possibilities for tuning both the porosity and sensitivity of the PDMS sponge [19]. A low cost technique for capacitive sensing was reported by the PDMS sponge obtained from the curing of the resin with simultaneous removal of yeast from the mixture. They studied the morphology and sensing action in detail highlighting the role of concentration and volume of yeast solution in the formation of sponge. The sponge morphology were also exploited for resistive sensing by using direct conducting entities like carbon based inks. Another method was reported by Tian et al. and group where sponge like carbon particles were obtained by laser scribed writing method for direct realization of resistive sensor. The sensitivity was achieved by the sponge like morphology of graphene with laser induced burning of it. By the laser lithography method adopted here, large area and pixilation enabled fabrication is feasible [112]. Another landmark in resistive sensing was made by Cho et al. wherein porous PDMS was fabricated using the physical method approach of proximity nanopatterning. This enables smooth and highly facile method of obtaining patterned arrays and sensors of any shape and dimension which is only lithography constrained. Further the porous structures were filled with conducting fillers and realized as resistive sensors. The highly ordered three dimensional morphology is unique for this approach as the phase mask is obtained with electron beam lithography and transferred onto PDMS by soft lithography. Though the process comes with a systematic and prolonged procedure, the periodic morphology is commendable and provides unique applications outside the sensing regime [107]. Chen et al. provided an omnidirectional sensitive resistive pressure sensor by using a cross design shape for CNT PU sponges. This was also integrated with a tribo generator to wipe out external noises [94]. Choi et al. presented a reverse process of incorporating the elastomer into a conducting 3D network of CNT synthesized from CVD process. The CNT sponge was immersed in the resin mixture and degassed and was cured with microwave heating for obtaining localized elastomer regions over the entire CNT sponge as CNT was more sensitive to the microwave rather PDMS. This resulted in thin strands of CNT covered with cured PDMS rather thicker strands giving better porosity and mechanical response [114]. The porous elastomer and sensing devices obtaining until were explored for integrated applications considering the large surface area available for charge separation and storage. Most resistive sensors have been realized with carbon based conducting moieties. Hence the high porosity made available to electrolyte-active carbon interface for efficient charge separation provide another regime towards self-powered devices. Sponge based supercapacitors paved way for integration of both the sensing element as well as energy storing combining the sensor with the supercapacitor fabricated from similar porous structures. The higher surface area renders better charge separation at the electrode–electrolyte interface giving capacitance in the mF range for powering the sensor giving an integrated supercapacitor sensor device [109, 121–123]. Song et al. integrated a micro supercapacitor over a pressure sensitive resistive PDMS sponge for realizing energy storage and sensing. These were further extended towards array fabrication by assembling several individual cells for large area sensing [124]. A similar approach was illustrated by Song et al. wherein they used sugar lumps for PDMS sponge fabrication and implemented a supercapacitor integrated resistive sensor. Their device was powered with the capacitor device attached to one side of the resistive sponge [109]. Thus the sponge based devices enabled both cost effective pressure sensor fabrication along with possible integration towards self-powered devices.

### Textile and thin film based sensors

**Fig. 7:**
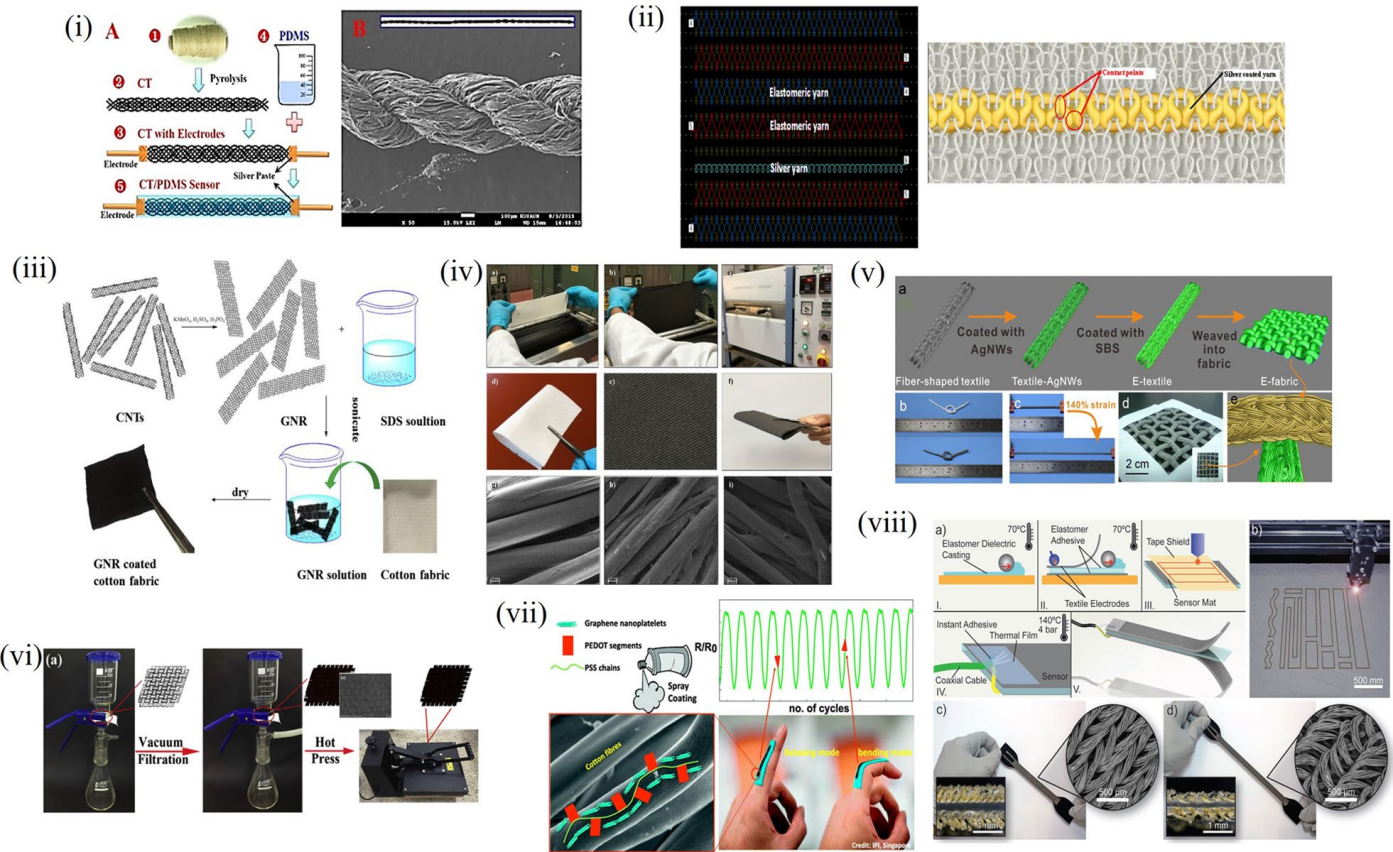
Textile based sensing platforms (i) Pyrolysis assisted CNT-PDMS sensor (Reproduced by permission from [125].*Copyright 2017, Nature*) (ii) Elastomer yarns supporting silver coated yarn giving stitched textile like sensor (Reproduced by permission from [128] *Copyright 2013, MDPI*) (iii) Cotton fabric made conductive by dip-dry in CNT-Graphene solution (Reproduced by permission from [132] *Copyright 2018, Elsevier*) (iv) Large scale fabric sensor fabrication with padding and rolling and uniform heating for flexible sensor (Reproduced by permission from [129] *Copyright 2017, American Chemical Society*) (v) Silver NW coated fibre yarn coated with SBS elastomer for flexible and stretchable sensor (Reproduced by permission from [134] *Copyright 2018, Springer*) (vi) Vacuum infiltrated fabric with graphene oxide (GO) and reduced to rGO by hot press giving area scalable and uniform heating (Reproduced by permission from [131] *Copyright 2017, Elsevier*) (vii) Spray coated PEDOT on fabric (Reproduced by permission from [130] *Copyright 2017, Elsevier*) (viii) Textile electrode elastomer dielectric based sensor with laser assisted shape and size control (Reproduced by permission from [140] *Copyright 2017, Wiley*)

Another advancement in achieving an all flexible and stretchable pressure/strain was towards incorporating conducting fillers directly onto textiles and woven yarns in the form of conducting threads [125–134] and as thin films and meshes encapsulated over patterned elastomer [80, 85, 135–137]. The sensing mechanism revolves around the orientation of the contact points in the yarns giving distinct response for tactile as well as pressure stimuli. When elongated or stretched, the contact point density decreases increasing the net resistance and with compression, the contact point density increases decreasing the resistance. A similar sensing mechanism follows for a twist or folding of the textile allowing it to retain the textile property and provide sensing action. The yarn are made conductive in many ways from weaving the elastomeric conductive fiber with the stretchable textile or by dip-dry method of absorbing the conducting entity within the cloth yarns or by a hot press reduction of graphene material after the cloth is dipped in the solution. These provide large area scalable fabrication of textile sensors which can be further incorporated into daily wear apparels for real time monitoring of human activities. When acquainted with wireless embodiments and circuitry, wireless tracking and monitoring is also feasible. These are elaborated in Fig. 7. Paradiso et al. showed sensors knit into textiles for wearable health monitoring systems. They also enabled an off the shelf data collection and diagnosis platform with data transmission with another integrated data acquisition system. This distributed sensing system also defines localized functions for the sensors with distinct sensing operations defined for each. Thus a complete health monitoring and remote supervision of the patient was proposed with this conducting fabric woven knit system [138]. Merritt et al. recommended use of oppositely aligned parallel plates attached to elastic and fabric which when pulled apart align themselves to form an overlapping area determining the capacitance change by stretch. They also included instrumentation circuits along with the sensor device for signal refinement and noise cancellation within the fabric for respiratory monitoring applications [127]. Atalay et al. showed knitted sensors elastomeric yarns with different linear yarn density to create interlock based structures, because the interlock structure has the highest dimensional stability among the basic weft knitted structures. This characteristic enables the creation of more reliable sensors in terms of repeatability using elastomeric woven knits and silver nanoparticle coated knits. This enables precise tactile sensing by the stretchability of the silver coated knits between the elastomeric knits [128]. While knitting was a found solution, Arogbonlo et al. used commercially available conducting fabric of tin/copper over silver coated nylon fabric along with proper device isolation by encapsulation of the pressure sensitive layer [139].

A machine facilitated stitching process was carried out by Gioberto et al. to obtain two sensors in a single machine operation as the top and bottom threads were isolated by an intermediate fabric layer as part of the stitching process. Here the resistance per unit length of the thread is counted; upon a stretch, resistance decreases giving a sense action [133]. Another configuration was to embed cotton fabric with conducting moieties of graphene by dip-dry procedure as elaborated by Gan et al. using graphene nanoribbons synthesized from MWCNT and dispersing them in distilled water using Sodium dodecyl sulphate (SDS) surfactant. The cotton fabric is then dipped in the dispersion and dried repeatedly to obtain sufficient resistance for effective sensing [132]. As batch processing and large scale production is possible via the chemical synthesis route, arrays and sensors for different applications depending on the resistance is also possible. Another advancement was made by obtaining textile electrodes for flexible sensors. The elasticity of elastomer is extended to the electrode via a laser imprint method and electrical connections are made via thermal bonding for robust sensing and better baseline capacitance to overcome parasitic capacitance effects as reported by Atalay et al. [140]. Graphene based conducting moieties by virtue of their hydrophobic nature are difficult to disperse in water. Ren et al. obtained good conductivity in fabric by dipping the fabric initially in GO solution and further reducing it to rGO by a hot press procedure for tactile sensing applications [131]. Cotton fabrics are reported to have a mechanical behavior similar to elastomers. However they have an elongation at break of less than 50%. Zahid et al. showed the use of PEDOT:PSS binder with graphene nanoplatelets along with mercerization of cotton fabric which is simple sodium hydroxide treatment for retaining the mechanical properties of the fabric and imparting them with better conductivity and robust nature [130]. The stretchability factor of fabrics were further improved by coating them after making them conductive with a thin layer of elastomer securing the conductivity as well as making them stretchable. Chen et al. used pristine cotton fibers and coated them with silver nanowires and these were further made elastic using SBS polymer coating. These resistive fibers were then sewn into textiles of desired dimension and shape enabling large area pixelated sensing [134]. Thus the missing link of integrating conformity of sensor devices and making them wash proof was executed by implementing textile based sensors. Their stretchability was however critical and was enhanced by adopting methods to maintain the potential hydrogen (pH) level of processes within their tolerance limits by alkaline treatments and adopting elastomer coatings etc. Thin films of piezoelectric materials were also in the lime light for their stability and sensitivity. Silicon based MEMS technology dominated the thin film based sensor fabrication techniques until when Wisitsoraat et al. came up with a non-silicon based MEMS fabrication of thin film diaphragm based sensing. Glass substrate was chosen and layered structures of patterned Au/Cr/AlN were deposited on a sacrificial photoresist layer with a final layer of ITO sputtering at different oxygen contents. Piezoresistive behavior of ITO was then measured for the applied pressure variations [141]. Yi et al. reported on another piezoelectric thin film based sensor using PVDF thin films clamped at two ends. Laser assisted deflection study of the light from the buckling of the film was studied for strain measurements [142]. Though single cell devices were fabricated using thin films, Yu et al. demonstrated a patterned sensor array using patterned collector assisted electrospun PVDF films and further dipping in polyaniline (PANI) solution for forming a patterned PVDF-PANI film resulting from oxidative polymerization of PANI over the PVDF fibrous structures. The large area patterned array was then employed in strain measurements [143]. Lipomi et al. demonstrated thin films of CNT patterned over ecoflex and electrical contact extended using liquid metal encapsulations. The CNT is spray coated initially over bare relaxed PDMS film. These are then stretched along to align and buckle up the CNTs. Upon bidirectional stretching, the CNT become buckled up in all directions enhancing the uniformity of conductivity in all directions [144]. Gupta et al. introduced a novel silicon thin film piezoelectric based sensing by using a dicing before etching technique for making a thin film of silicon over which the piezoelectric material of P(VDF-TrFe) is coated. The thin Si membrane allows for easy strain and bending applications giving higher sensitivity [145]. Thus textile and thin film based sensing mechanism provided another regime of device fabrication opening up further options for application specific architecture.

### Printed sensors

**Fig. 8:**
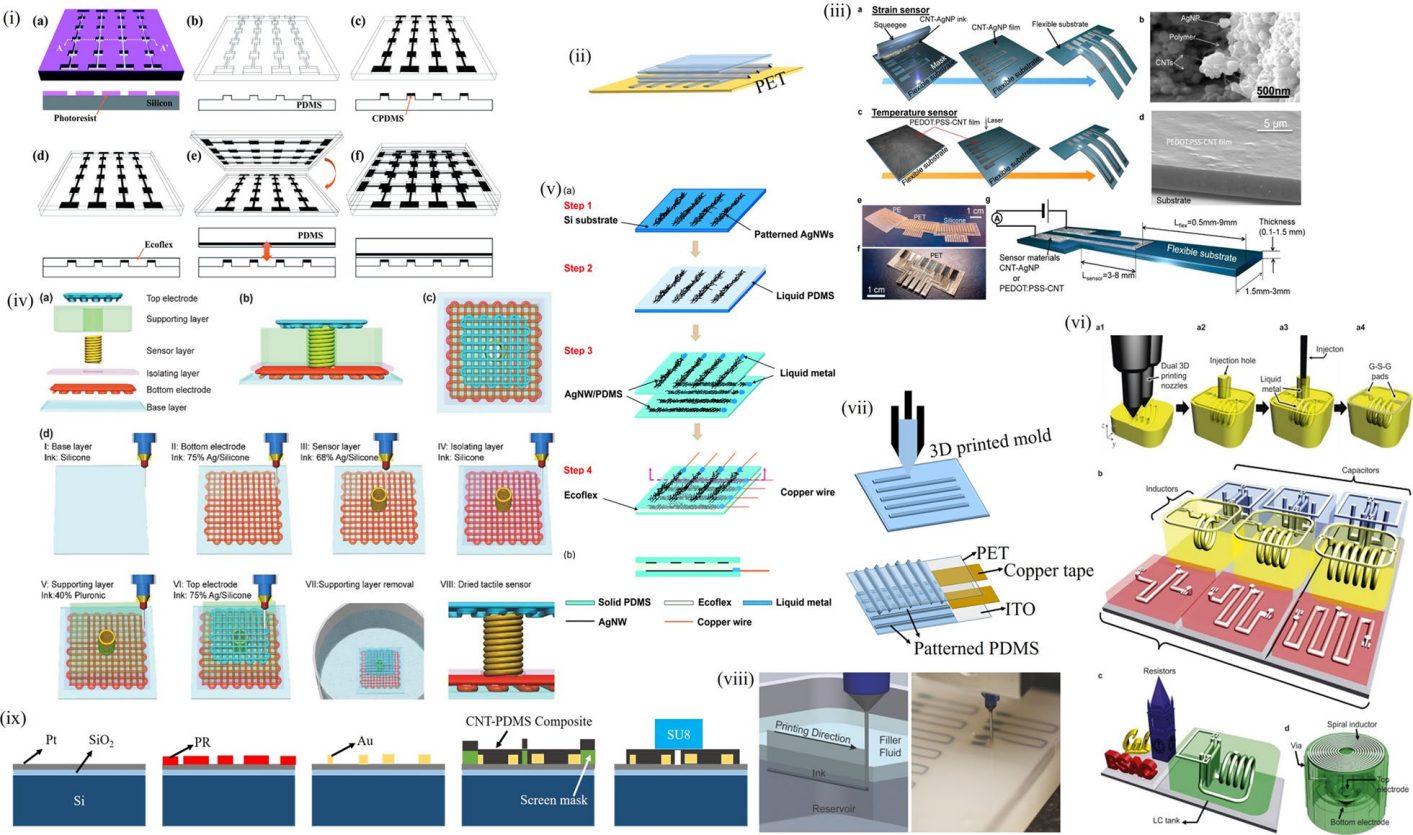
Printed technology in sensor fabrication (i) Micropatterned CNT upon microstructured PDMS layer (Reproduced by permission from [165] *Copyright 2014, Royal Society of Chemistry*) (ii) Direct printing of electrode layers. (iii) Whisker pattern printing of electrode (Reproduced by permission from [167] (*Copyright 2014, American Chemical Society)* (iv) 3D printed layer by layer sensor device fabrication (Reproduced by permission from [187] *Copyright 2017,Wiley*) (v) Liquid metal printing and consecutive PDMS curing for stretchable printed sensor (Reproduced by permission from [170] *Copyright 2014, Royal Society of Chemistry*) (vi) Full printed integration of sensor and electronic circuitry (Reproduced by permission from [188] *Copyright 2015, Nature*) (vii) Mold fabrication for microstructure patterning of PDMS. (viii) Embedded 3D printing of ink in elastomer (Reproduced by permission from [189] *Copyright 2014, Wiley*) (ix) Mask fabrication for composite sensor elements

Lithography techniques were largely used for satisfying the emerging needs of automation and large area processing of devices until printing was found to be better for the wide range of materials and the feasibility possible with it [146]. The different layers of a sensor have been printed successfully, the electrodes and the sensing element including a complete e-skin [147–157]. The MEMS approach was regenerated with the help of screen printing for obtaining the air gap or the diaphragm precisely over metal electrode layers. The composite paste was optimized for optimal thickness and cured for completion of process. Earlier printing techniques were employed in printing of electrodes alone utilizing one or more of the above mentioned methods for the sensing element. Transistor based sensors were developed with microstructure patterned PDMS enabling sensing action from the air voids in the microstructures of the dielectric layer. Other completely printed sensors were from the piezoelectric layer upon flexible substrate like polyimide (PI), PET etc. 3D printing however provides maximum feasibility for the different choice of materials that can be incorporated within a single sensing element for obtaining hybrid sensing in an on the go fabrication process. Varied printing techniques exist and are utilized for their minimum feature size specificity. For nozzle discharge printing techniques like 3D printing, the nozzle diameter defines the feature size possible. In 3D printed devices, each layer is printed like the top and bottom electrodes, sensing element, support sacrificial layer with final removal from the base layer. These also provide choice of varied elastomer materials for their critical softness and Young’s moduli. This has an added feature of low material wastage and large area scalable fabrication giving cheaper options. Embedded 3D printing enables simultaneous encapsulation of the filler ink within the elastomer substrate allowing a distributed phase of liquid filler ink and room temperature curing giving a highly inter layer compatible sensor configuration. Elastomers like Dragon skin 30, Ecoflex Smooth 00-00 provide room temperature curing and mutual compatibility with electrode materials giving complete printed device. While printing provides precise control over dimensions and thickness, synthesis of optimized ink is critical for these. The major challenge in them is the proper ink stability that can provide required rheology for ease of device manufacture exploiting the utility of the printing technique. Printing in itself is a very broad area with varied printing options made available by virtue of the specific necessities of applications. These come in the form dimensions, resolution, limit of stretchability and conductivity and substrate constrains. Thus a printing technique is adopted for the particular application like screen printing, inkjet printing, roll to roll printing, grauvure printing etc. Figure 8 below briefs these techniques. Diaphragm based sensing found limited use for the constraints of MEMS technology. However, these were renewed with the advent of screen printing of diaphragms for large scale miniaturized applications as depicted by Sippola et al. using a ceramic capacitive pressure sensor. The thick cavity was designed with a systematic layer by layer deposition of dielectric and sacrificial layers for securing the inner layers and final encapsulation of the device with thick layer screen printing technique [147]. In the mean while 3D printing emerged as an automated printing technique for the computer aided design (CAD) designing involved in it, it enabled a facile approach for any design and dimensionality to be fabricated at low cost and time. These were targeted for prosthetic limb applications with 3D freedom of movement. Thus a distinct 3D performance analysis was required. This was facilitated by the fabrication of different electrodes for each direction and each paired with a common electrode. The sensing element is sandwiched between the distinct electrode pairs for distinguishing the 3D sensing actions [158]. Gerlach et al. used screen printing for gait analysis applications by using PDMS-MWCNT and P(VDF-TrFe) composites for insole device array installation and real time monitoring. The gait pattern help distinguish between diseases like diabetes, obesity, peripheral neuropathy or combinations of these. The array fabrication with screen printing helps easy fabrication for the desired sole size and shape giving customized sensor arrays [159]. They further modified the same by allowing for a pressure concentrator [160]. Hassinen et al. elaborated upon a roll-to-roll printed organic transistor based active matrix backplane for use and throw medical applications. Varied printing processes were utilized for optimal array matrix production. Like the top foil was developed using inkjet printing and screen printing for defining the electrodes. The spacer was printed using inkjet printing with intermediate foil treatments for better film adhesion and patterning [153].While several combinations of printing techniques were being studied, Noguchi et al. elaborated the organic transistor fabrication using an all inkjet printed back plane for pressure sensing wherein they adopted a partition defined device dimension for ensuring film thickness uniformity over the whole substrate [161]. Daniel et al. demonstrated a fully printed sensor for both pressure and acoustic sensing. They combined printing and lamination methods for device fabrication. A polyimide sheet substrate is taken and the P(VDF-TrFe) composite was patterned using inkjet printing that is also compatible with roll to roll printing process for scaling up the fabrication [162]. Narakathu et al. studied the possibilities of using both screen printing and gravure printing towards developing a fully flexible pressure sensor by gravure press printing the silver electrode and screen printing the dielectric PDMS layer [163]. They also extended the device fabrication towards an array of 4 × 4 by gravure printing bottom four electrodes and then the screen printed PDMS layer with a subsequent gravure printed top silver electrodes on PET flexible substrate. Ando et al. elaborated another all inkjet printed strain sensor by using a water based commercially available silver conducting ink on PET sheet. They fabricated electrode patches of track width and pitch 200 and 300 microns respectively for varied track lengths and found the track resistance to vary as the Ohms law [164]. Woo et al. proposed an all elastomer sensor with micro contact printing technique using CNT-PDMS composite as the electrode and ecoflex as dielectric layer. Array fabrication was very facile for the large area scalability and less time for fabrication. However the sensitivity was limited by the composite resistance. Though the whole device fabrication was easy with the approach, the alignment of electrode layers defines the areal capacitance and resolution [165]. A multi modal sensor was illustrated by Harada et al. using a combination of mask assisted printing of the CNT-PDMS composite for temperature sensing and screen printed electrode layers of silver for strain sensing [166]. This sensor was additionally attached with an SU-8 bumpy layer enabling for three dimensional strain sensing which is peculiar for the bump layer. Screen printing allows for different kinds of composite materials to be printed with least contamination as the mask alone needs to be changed. Harada et al. showed this by printing PEDOT:PSS- CNT composite for a whisker shaped electrodes for arrayed patterns for e-skin based pressure detection. The whisker shape for the electrodes provide change in resistance for displacement of electrodes. As the whisker are more flexible than the sensor body, slight movement or displacement can also be detected with good sensitivity [167]. Screen printing shows promising scalability of sensor fabrication; however, the curing process can be altered to adopt a speedy fabrication. Bessonov et al. used photonic curing of graphite conducting ink for its faster curing rather conventional thermal processes [168]. Another approach executed by Watanabe et al. was screen printing of piezoelectric poly(amino acid) films over sintered silver paste electrodes upon a flexible substrate like polyimide. The electrodes were sintered for drying while the piezo poly(amino acid) was dried at room temperature for 24 h. The piezoelectric behavior was confirmed from the dual nature of voltage developed across the film with displacement of the dipole moments in them. A track mat was also designed and fabricated using the approach for the large area scalability of the printing technique [169]. The screen printing was also further used by Yao et al. for developing a multifunctional capacitive sensor for sensing pressures up to 1.2 MPa; silver electrodes were patterned on Si wafer initially, liquid resin cured over it provided good adhesion of the electrodes with the PDMS which enabled easy peel off of it with the electrodes giving free standing stretchable substrate for the electrode patterns. Ecoflex layer as the dielectric provided a completely stretchable sensing device with array pattern for large area pixelated sensing [170]. Yeom et al. took the printing techniques towards a commercial device sensing applications as the back plane transistor array for sensing action. They used the roll-to-plate gravure printing for transistor array fabrication defining the pixels of the array. A pressure sensitive rubber is then used between the source and ground terminals giving normal transistor action during a press event on the pressure sensitive rubber layer. Large area 18 × 18 pixel array was fabricated [171]. Thus with the alarming development in the field of printing technology, sensor device and array fabrication has reformed itself to a new genre and transplanted from the rigid wafer to a fully stretchable sensor device.

### Towards hands free sensing

**Fig. 9:**
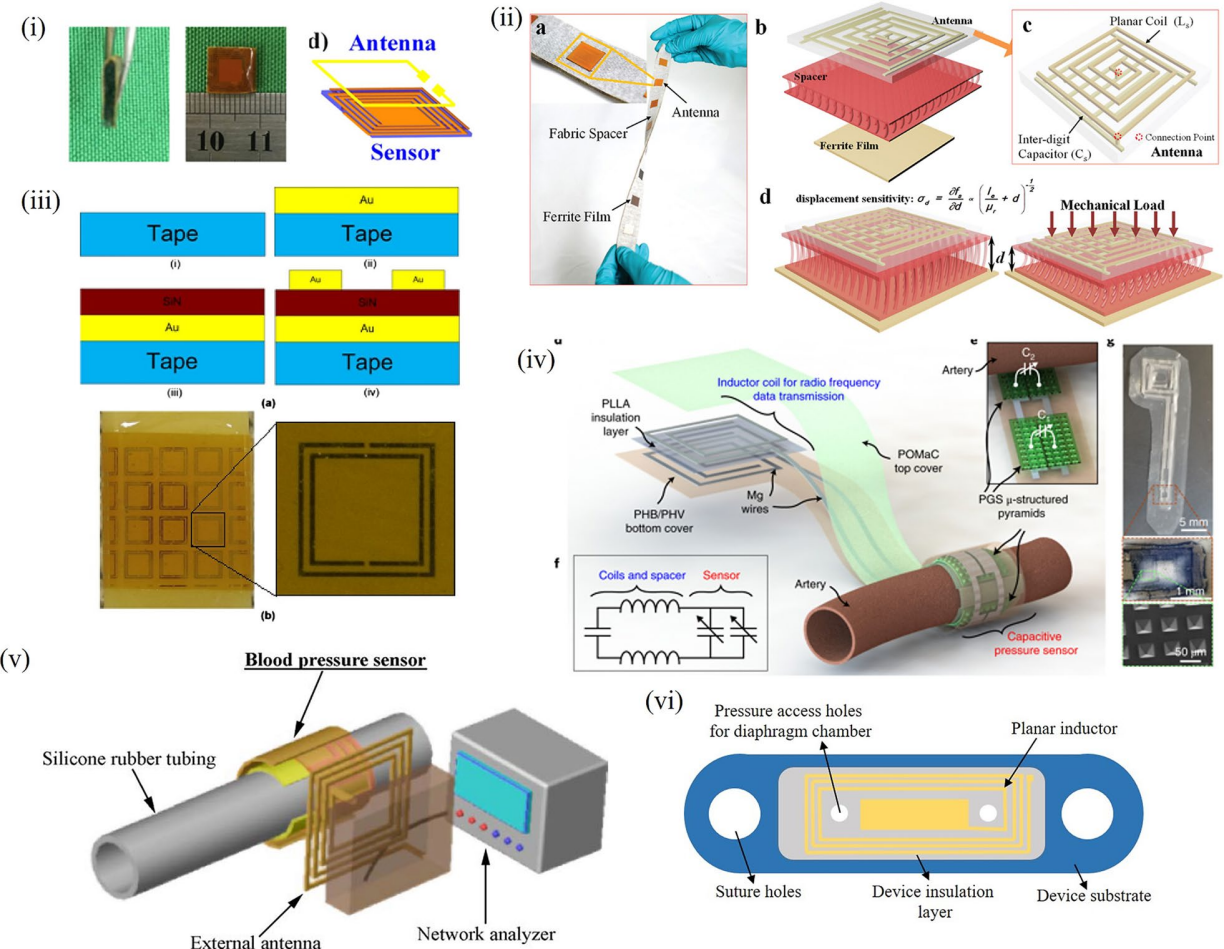
Wireless pressure sensors (i) GR/PDMS sponge based pressure sensor with attached LC circuit (Reproduced by permission from [190] *Copyright 2019,Nature*) (ii) Fabric spacer based capacitive sensor with a ferrite film and LC passive antenna (Reproduced by permission from [180] *Copyright 2019, Wiley*) (iii) Tape based sensor fabrication and final integrated antenna based sensor (Reproduced by permission from [176] *Copyright 2009, American Institute of Physics*) (iv) Biodegradable micropyramidal patterned sensor with RF data transmission (Reproduced by permission from [181] *Copyright 2019,Nature*) (v) Schematic of external in vivo pressure measurement (Reproduced by permission from [191] *Copyright 2005,Elsevier*) (vi) Diaphragm based wireless antenna integrated sensor

Sensors require a data acquisition system for data retrieval and presentation for further understanding. This makes the sensor wired to bulk systems. Raw data is seldom used for direct diagnosis and thus required filtering and signal processing done by electronic circuitry. This subsequent processing adds to the bulkiness of device. An effort towards reducing the bulkiness at the device level was to isolate the signal refining circuitry from the sensing part. Thus making them stand alone essentially requires a powering circuit along with data transmission for well refined useful data. Two data transmission strategies exist for data transmission of raw data from the sensor. DeHennis et al. elaborated on the passive telemetry mode of data transmission namely passive load modulation and resonant peak monitoring. An on-chip circuitry for a wireless system to actively modulate a reflected load on a coupled primary inductor defined the former approach while latter uses a wireless tank circuit (LC) through a local minimum in the phase of the impedance characteristics of a coupled primary inductor *vs* frequency plot. The resonant peak passive telemetry is used for wireless pressure sensing by a capacitive pressure sensor in a wireless LC tank circuit. They implemented a single chip double sided fabrication procedure with the diaphragm fabricated on one side and the antenna on the other. This configuration used a metal stack layer for establishing glass to metal and metal to silicon interconnection for complete device fabrication [172]. Amplifiers and filters form the basic constituents for these which are nowadays readily available. However, they occur in the rigid substrate which would require additional interfacing with the soft substrates. This is either implemented as transmitting the data through wireless communication systems or as a complete refining circuit interfaced with the sensor device. In either scenario a wireless transmission of raw data or the refined data is necessary. This thrust thus became a serious aspect when progressing towards miniaturization. It thus became a necessity for bringing about integrated devices housing all the refining circuitry for a reliable result from the sensing platform. A step towards this as seen in the microstructured elastomer section is the tribo generator function with sensing action procured from the microstructure of film. However, this does not house the signal refinement and data transmission for remote monitoring. These are demonstrated in Fig. 9. An effort towards agricultural produce monitoring was depicted by Keshri et al. in monitoring the produce quality by sensing the oxygen and carbon dioxide content in it. A printed circuit board (PCB) housing sensors for low and high carbon dioxide sensors along with communication chips are incorporated inside a shell structure and screwed along with it [173]. Intraocular pressure sensing became a probing cause to blindness has been a serious concern in the biomedical field. The disease requires continuous monitoring of the intraocular pressure to check for the progressive blindness that can be incurred by the damage to the optical nerves. Thus together to form a complete sensor package for ideal real time respiration rate measurement of the minimally invasive techniques were required at large for wireless real time monitoring of the pressure and thus it became necessary for the miniaturized device fabrication techniques to fit the sensor and transmission within the eye of the patient. Early works report a minimally invasive transmission designs for the tank circuit giving optimal sensing. Going wireless with pressure sensing has been realized with the MEMS technology itself using antenna for data transmission making the signal refinement at the receiving end. Chen et al. designed an implantable parylene based wireless pressure sensor for monitoring intra ocular pressures in glaucoma patients. A MEMS fabricated variable LC circuit facilitated the variable capacitance and inductance measurement and transmission using an external LC circuit. A deformable diaphragm chamber with respect to pressure changes provides a capacitive changes in the sensor [174]. With regard to biomedical applications, Fonseca et al. demonstrated complete in vivo monitoring of respiration with a wireless sensor implanted in a dog with good agreement of transmitted data and original data [175]. Sensor antenna design essentially consisted of a cavity bound by two capacitor plates or distributed capacitive structures which are resonantly interconnected with an inductance. A deflection of either the top or bottom sides of the cavity gives a change in the capacitance that causes a change in the resonant frequency of the passive LC circuit measured wirelessly through an external magnetic loop. Loop based antennas were replaced with split ring resonators (SRRs) realized with metamaterial structures for achieving higher quality factor and miniaturized designs. Melik et al. briefed this process with fabrication of SRRs on polyimide flexible substrate compared to conventional silicon rigid substrate with an integrated capacitive sensor. The sensing element was placed within the SRR dimensions giving an array fabrication. With applied pressure, the resonant frequency of the SRR changes giving a sensing action [176]. The same group further explored the wireless possibility by incorporating external horn antenna for transmission of the change in resonant frequency compared to probed measurements executed in [176]. An increased gap in the SRRs enables for increased capacitance of the device and also helps improve the sensitivity compared to coil structure employed previously [177]. Cheng et al. introduced the concept of multi-layer microfluidic reversibly stretchable wireless strain sensors. They provided a liquid metal based radio frequency (RF) antenna by microfluidic channel approach with a fluidic ground plane and fluidic patch for transmission of capacitive sensor data [178]. The sensor was powered with an integrated flexible printed circuit board with two flex to stretch interconnects and encapsulated in a PDMS localized stiff cell providing enhanced reliability and durability. The device could withstand strains up to 15% for safe operation. This limitation was encountered for the soft to rigid transformation mechanical property mismatch. Huang et al. demonstrated upon wireless transmission of hydration and strain sensors integrated with transmission circuitry realized as a layered polyimide copper serpentine structures. The thin layered structure allowed for good skin adhesion and real time monitoring of both the mechanical and dielectric changes in skin due to changes in the skin properties undergoing by induced actions [179]. Chen et al. elaborated a proof of concept wireless sensor at its miniaturized state by using patterned SBS elastomer and coil RF transmitter device. SBS was used for its minimum losses at higher frequencies of measurement compared to PDMS and PU but compromising on the cyclic capacitance stability. A maximum sensitivity was achieved for 50 micron track width for the 5-turn coil design with decreasing sensitivity for decrease in the track width [23]. With increasing demands of remote monitoring and diagnosis, wireless sensing has gained and continues to gain thrust on new designs and developments for sensing both in vitro and in vivo. Nie et al. recently designed a novel fabric spacer based wireless strain sensor using a loop antenna as top plate and a ferrite coated film as bottom plate. When an external pressure is applied, the fabric spacer undergoes a mechanical compression causing an inductance change which transduces to a detectable change in the resonant frequency with added advantage of the ferrite layer being providing high permeability improving the sensitivity and shielding the device from interference of conductive materials simultaneously [180]. The materials choice and transmission circuit design are critical for the power requirement and noise effects on the sensor performance. Biomedical implant real time monitoring would require biocompatible materials for both data transmission and sensing. In cases of post operation care where the implant monitoring is critical and short termed, removing the sensor can lead to complications. In such cases a biodegradable sensor serves the purpose of both real time monitoring as well as self-degradation after use. Boutry et al. facilitated this achievement by designing an all biodegradable sensor wrapping the artery for blood flow monitoring with wireless monitoring through a loop LC circuit. Capacitive sensing was implemented using micro structured pyramidal elastomer layer and antenna was attached to it using polyhydroxybutyrate/polyhydroxyvalerate (PHB/PHV) bottom layer and Mg wires for the antenna and poly(lactic acid) (PLLA) as insulator and poly(octamethylene maleate (anhydride) citrate) (POMaC) cover layer. The pyramidal structures enable capacitive sensing while the antenna transmits the change in capacitance inductively. A receiver at the diagnosis end collects the data through a loop antenna. After a few months of observation, the biodegradable sensor device undergoes degradation having served the whole purpose [181]. Going wireless thus essentially involved using a sensing element or architecture demonstrated in the previous sections and integrating them with either RF or SRR frequency dependent structures. The SRR structures provide compact designs for the antennas enabling device miniaturization as well as array and batch processing.

## Conclusion

This manuscript provides a brief insight into the development of sensing devices and their evolution over configuration and fabrication technologies. The MEMS technology had an initial bloom period which saturated for their miniaturization limitation and process complexity. With surface conformity being a necessity, the rigid materials which defined the MEMS technology failed to accomplish and thus elastomers and polymer emerged. These with their tunable mechanical properties and process feasibility have almost replaced the MEMS regime. However, recent studies in PDMS still preserve a few of their applications. A systematic study of the evolution shows an increase in the complexity and successive progress giving better performance. Recent trend rests in the permutations and combination of already existing process methodologies towards obtaining large area scalable array of sensors for distinct sensing capabilities. Multimodal sensing with single entity has also been found to be promising towards cost effective fabrication for their multi parameter sensing capability. Printing technologies are advancing towards a bigger leap of obtaining a fully printed sensor. This has been achieved by the optimization of inks for different layers and also enables simultaneous encapsulation for a complete device. With the advent of flexible circuits realized with the existing technologies for obtaining flexible electrodes and interconnects integrating sensors with electronics circuits for data transmission to go hands free operation has also emerged. This enables a sensor to be devised as a stand-alone system being self-powered and wireless data transmission which are then further refined and analyzed for real time monitoring purposes as well.

## Data Availability

The review is based on the published data and sources of data upon which conclusions have been drawn and can be found in the reference list.

## Abbreviations

MEMS: micro electromechanical systems
PDMS: polydimethylsiloxane
SU-8: a negative epoxy based photoresist
CNT: carbon nanotube
MWCNT: multiwall carbon nanotube
NEMS: nano electromechanical systems
SBS: poly(styrene–butadiene–styrene)
PU: polyurethane
kPa: kilo Pascal
MPa: mega Pascal
rGO: reduced graphene oxide
PEDOT: PSS-Poly(3,4-ethylenedioxythiophene) Polystyrene sulphonate
NW: nanowire
SDS: sodium dodecyl sulphate
GO: graphene oxide
pH: potential hydrogen
PVDF: polyvinylene diflouride
PANI: polyaniline
PI: polyimide
PET: polyethylene terephthalate
3D: three dimensional
CAD: computer aided design
h: hours
LC: electronic circuit composed of inductor and a capacitor
vs: versus
PCB: printed circuit board
SRRs: split ring resonators
RF: radio frequency
PHB/PHV: polyhydroxybutyrate/polyhydroxyvalerate
PLLA: poly(lactic acid)
POMac: poly(octamethylene maleate (anhydride) citrate)
